# The Venus score for the assessment of the quality and trustworthiness of biomedical datasets

**DOI:** 10.1186/s13040-024-00412-x

**Published:** 2025-01-09

**Authors:** Davide Chicco, Alessandro Fabris, Giuseppe Jurman

**Affiliations:** 1 Università di Milano-Bicocca & University of Toronto, Toronto, Canada; 2 Max Planck Institute for Security and Privacy, Bochum, Germany; 3 Fondazione Bruno Kessler, Trento, Italy

**Keywords:** Biomedical data quality, Data trustworthiness, Data documentation, Medical data, Health informatics, Bioinformatics, Trustworthiness, Trustworthy data, Computational biology, Cheminformatics, Medical text, EU AI Act, Datasheets for Datasets, Kaggle

## Abstract

Biomedical datasets are the mainstays of computational biology and health informatics projects, and can be found on multiple data platforms online or obtained from wet-lab biologists and physicians. The quality and the trustworthiness of these datasets, however, can sometimes be poor, producing bad results in turn, which can harm patients and data subjects. To address this problem, policy-makers, researchers, and consortia have proposed diverse regulations, guidelines, and scores to assess the quality and increase the reliability of datasets. Although generally useful, however, they are often incomplete and impractical. The guidelines of *Datasheets for Datasets*, in particular, are too numerous; the requirements of the *Kaggle Dataset Usability Score* focus on non-scientific requisites (for example, including a cover image); and the *European Union Artificial Intelligence Act* (EU AI Act) sets forth sparse and general data governance requirements, which we tailored to datasets for biomedical AI. Against this backdrop, we introduce our new Venus score to assess the data quality and trustworthiness of biomedical datasets. Our score ranges from 0 to 10 and consists of ten questions that anyone developing a bioinformatics, medical informatics, or cheminformatics dataset should answer before the release. In this study, we first describe the *EU AI Act*, *Datasheets for Datasets*, and the *Kaggle Dataset Usability Score*, presenting their requirements and their drawbacks. To do so, we reverse-engineer the weights of the influential Kaggle Score for the first time and report them in this study. We distill the most important data governance requirements into ten questions tailored to the biomedical domain, comprising the Venus score. We apply the Venus score to twelve datasets from multiple subdomains, including electronic health records, medical imaging, microarray and bulk RNA-seq gene expression, cheminformatics, physiologic electrogram signals, and medical text. Analyzing the results, we surface fine-grained strengths and weaknesses of popular datasets, as well as aggregate trends. Most notably, we find a widespread tendency to gloss over sources of data inaccuracy and noise, which may hinder the reliable exploitation of data and, consequently, research results. Overall, our results confirm the applicability and utility of the Venus score to assess the trustworthiness of biomedical data.

**Supplementary Information:**

The online version contains supplementary material available at 10.1186/s13040-024-00412-x.

## Introduction

While the “garbage in, garbage out” (GIGO) statement is almost 70 years old [1], the awareness of the critical importance of data quality in computing dates back much earlier, to the point that even Charles Babbage himself is blatantly explicit on the issue ([2], page 67). The outcome of the use of unreliable input data ranges through different levels of output inconsistencies which, depending on the background domain, may reflect in quite harmful consequences [3–8]. Despite the wealth of scientific work tackling the problem (also through large initiatives such as FAIR [9] or MAIDA [10]), a shared and effective solution is still far from being acknowledged, and the current artificial intelligence (AI) revolution has contributed to worsening the situation [11], mainly due to the widespread diffusion of the generative paradigm.

A lot is at stake when the involved domain is healthcare and a rich scientific literature is available discussing the diverse aspects of such complex theme, together with several interesting reviews aiming at providing a broader perspective [12]. Leveraging from several real-world examples [13] and projects [14], the level of awareness of the need for data quality and trustworthiness assessment has been growing in the biomedical community. Such novel line of work has led to both theoretical and practical advancements, that is, the fact that the quality of clinical data ought to be constantly assessed and reassessed [13] and the implementation of new interesting practical tools [15] supporting such assessment. Notably, even the overall methodology has improved, adopting (meta)data driven approaches for the inspection of quality [16, 17].

Biomedical data can come in different format: medical images, genomics and proteomics, drug and chemicals data, medical text, electronic health records (EHRs), and others. Within this scenario, so far EHRs play a prominent role also when dealing with quality, as evidenced by the abundance of publications in the literature [18]. Indeed, many different aspects are covered: as major issues we list the minimal consistency and potential generalizability in the methods used to assess EHR data quality [19], calling for the installment of automatic, scalable and flexible guidelines to improve the efficiency, transparency, comparability, and interoperability of data quality assessment [20], also tailored and automatized [21] for specific secondary used in research [22]. Stemming from such needs, suites of practical guidelines [23] and *ad-hoc* quality scores for EHRs have been proposed – further contributing to the discussion we will unfold hereafter [24].

Note that not even the definition of data quality is univocal, although steps in this direction have been taken [25], and the heterogeneity of the data types in health and life sciences in general (electronic medical records, biomedical images, omics sequencing, etc.) represents an additional level of complexity. Last but not least, adding insult to injury, even the procedures aimed at preprocessing the raw data or the tools used to perform such transformation may inject unwanted noise or artifacts, further degrading the original quality, as in the anecdotal case of gene names mistreated by Microsoft Excel [26–28].

It is not surprising that the core of the data quality and trustworthiness assessment lies in the identification of the essential characteristics shaping a well-formed dataset. In the last few years, such a goal has led several research groups worldwide in building different quality scores collecting some of the aforementioned characteristics, attributing to each of them a quantitative value for a given dataset, and finally obtaining a single figure as a weighted cumulative sum of all the associated marks. The different published scores target for instance the different nature of the data, or focus on specific aspects of the data quality. As a result, a wide landscape of scores are currently available to the community of data scientists, from minimal with few items to enormous including tens of questions and from specific and oriented to single data type to quite broad and general instead. Unfortunately, using most of them for the quality assessment of datasets in life sciences is not feasible: large scores include too many negligible items not providing actual hints about quality and they are unpractical to be quickly filled, while small scores cannot properly encompass the rich diversity of the different nature of datasets in the healthcare domain.

Contributing to this literature stream with the aim of amending the above cited drawbacks, we propose the novel Venus score as an effective compromise between the need to intercept all the critical data quality and trustworthiness elements, the flexibility to adapt to the different healthcare data types and the usability of the introduced tool for the data scientists, thus obtaining a ten-item questionnaire. After a detailed overview of the current state-of-the-art of data repositories, existing quality scores and documentation frameworks, along with their pros and cons, we proceed with a thorough discussion of all the ten Venus items. Next we test the Venus score on twelve life science datasets belonging to six different categories, later concluding with a discussion of the results, that seem promising enough to promote the adoption of the new proposed tool by the data science for health community.

### Data repositories, search engines, and aggregators

Several online data repositories exist nowadays with different best practices for data curation, management, and documentation. Figshare [29], Zenodo [30], and the University of California Irvine Machine Learning Repository [31] are websites where anyone can upload and release a dataset and from where anyone can download datasets, of any type and on any subject. These three general-purpose data repositories give the possibility to associate a digital object identifier (DOI) code with a dataset, before release. Figshare, moreover, contains supplementary information on articles published in several biomedical informatics journals.

Kaggle, on the other hand, is a company and an online platform mainly hosting data science competitions, where users can use machine learning and statistics to solve scientific or technological problems [32–34]. Kaggle also gives the opportunity to download and publish datasets, and today, it contains around 330 thousand of them. We will talk more about Kaggle in the next paragraph (“Kaggle Dataset Usability Score”). Another source of datasets is Hugging Face [35], which is a company and a centralized web service hosting software code repositories and approximately 150 thousand datasets, especially for natural language processing (NLP) tasks.

Some websites, on the other hand, serve as aggregators of other online data repositories. This is the case of re3data [36], of Google Dataset Search [37], and of the GAAIN network data [38, 39]. The former two are search engines that allow users to find datasets on several platforms and repositories (including the ones mentioned above), and the latter one is a website containing links to other 66 websites having data on mental health that can be requested by anyone.

Web containers of data repositories can also be specific to particular biomedical data types or fields. A huge number of bioinformatics and genomics datasets, for example, can be found on Gene Expression Omnibus (GEO) [40], ArrayExpress [41], Sequence Read Archive (SRA) [42], and the Cancer Genome Atlas (TCGA) [43]. Researchers interested in medical images can find multiple datasets on the Cancer Imaging Archive (TCIA) [44, 45], while researchers interested in electrical biosignal data such as electroencephalography (EEG) or electrocardiography (ECG), can resort to PhysioNet [46]. The PhysioNet resources include physiologic data of intensive care unit (ICU), such as the MIMIC datasets [47, 48], too. Surveys and reviews comparing medical datasets can also serve as a good resource for finding public data of good quality [49, 50].

Even if a huge number of biomedical datasets are openly available online, they are rarely accompanied by a score that can summarise their trustworthiness and quality. Some studies introduced indexes and scores representing the technical quality of the dataset analyzed: we describe them in the next paragraph.

### Data quality scores

Multiple biomedical research teams have proposed data indexes and scores in the past. Michele Salati and colleagues [51], for example, released the European Thoracic Aggregate Data Quality score, that can be used to estimate the technical quality of data on lung resections. This coefficient measures the cumulative data quality of a dataset, and consists of sixteen factors that capture the completeness and the reliability of the dataset analyzed. Completeness looks at the proportion of null or blank data for specific features, while verifying if the dataset overcomes nine quality checks defined by the authors. One of these query checks, for example, verifies if the outcome of a patient at discharge is *died* and their outcome at thirty days is *alive*.

Regarding female health, Georgina Jones and coauthors [52] developed a study where they assessed the quality of data collected through questionnaires filled by women suffering from endometriosis. Their assessment involved questionnaire validation through secondary factor analysis, internal reliability consistency, descriptive statistics of the data including skewness, data floor and ceiling effects, and corrected item to total correlations.

Moving from hospitals to tech companies, it is worth mentioning the Data Quality Toolkit proposed by IBM [53]. This tool, seemingly available within the IBM technology platforms, characterizes the technical quality of the dataset employed in a training phase of a machine learning project. The Data Quality Toolkit of IBM consists of ten technical factors (class overlap, label purity, class parity, feature relevance, data homogeneity, data fairness, correlation detection, data completeness, outlier detection, and data duplicates) that can give an overview of the technical aspects of the dataset analyzed.

Data quality scores were also designed for administrative and demographic purposes. This is the case of the Irish Data Quality Index [54] and of the Open Data Toronto Data Quality Score [55–57]. The former is an index developed and implemented within Ireland’s Health System, where clinical records of patients get higher marks the more complete they are, while clinical records with missing information get lower marks. The latter was designed and implemented at the City Hall of Toronto, and evaluates each available administrative dataset based on its freshness, metadata, accessibility, completeness, and usability.

We also mention the RECORD Statement [58], which is a set of questions on the completeness of medical studies that evaluates scientific articles. Authors suggest to use the thirteen questions of the RECORD statement to verify if pivotal information regarding a specific study is present in title, abstract, introduction, methods, results, and discussion section of its associated publication. A variant of RECORD for pharmacoepidemiology studies was released at a later time [59].

The FAIR community, moreover, proposed its own scores for assessing the fairness of datasets and software: the FAIR Aware questionnaire [60] and the Fairness score [61]. As the name suggests, the former states the levels of awareness on the findability, accessibility, interoperability, and reusability (FAIR) requisites that a dataset should have. The latter, instead, uses automated software mainly to evaluate the presence of the fields of the metadata of a dataset.

These data quality scores, indexes, and coefficients attempt to quantify the level of technical reliability of medical datasets, but do not say much about their quality and trustworthiness. We therefore felt the need to design a new score for assessing biomedical data quality scientifically, by considering the founding principles of the recent *Artificial Intelligence act* of the European Union [62, 63], the guidelines of the *Kaggle Dataset Usability Score*, and the recommendations of *Datasheets for Datasets* [64]. We describe these three frameworks in the next paragraphs.

### European union artificial intelligence act

The AI Act is a European regulation laying down harmonized standards for AI systems [62, 63, 65]. High-risk AI systems, including the ones deployed in healthcare settings, must comply with dedicated provisions, with special attention to data and its governance. In particular, providers and deployers of medical AI must demonstrate that training, validation, and testing datasets meet the quality standards described in different sections of the act, especially Article 10. The technical documentation accompanying a high-risk AI system should describe the data, including its collection process and purpose, its preparation, and possible biases that may lead to discrimination, along with a discussion of data accuracy and representativeness with respect to the specific geographical, contextual, and functional setting within which the high-risk AI system is intended to be used. The European Parliament published the AI Act in its final version on 12th July 2024, and it came into force on 1st August 2024. Given its novelty, none of the currently existing frameworks for data quality assessment incorporate its principles and provisions; we fill this gap by proposing our Venus score, which we will describe later.

### Kaggle Dataset Usability Score

The Kaggle Usability Score is a coefficient invented by the Kaggle team to quantify the completeness, credibility, and compatibility of a dataset [66]. It ranges from 0 to 10, where 0 means no usability and 10 means maximum usability.

Kaggle revealed the eleven fields of this score but never disclosed their weights [67, 68]. We attempted to deduce these weights by considering datasets which had one single field missing, and deducing its value by subtracting the dataset’s score from the total.

For example, we noticed that the *Fitbitdata* dataset [69] has the *Column Description* field missing, and that the total score for that dataset is 9.41. We therefore estimated the weight of *Column Description* to be $10 - 9.41 = 0.59$, and we observed that other datasets lacking that field had the same score. Similarly, we checked the dataset called *A Hotel’s Customers Dataset* [70]: its usability score is 8.82 and the only score field absent is *File Format*. We therefore can deduce that its weight is $10 - 8.82 = 1.18$, and we observed the same value for that field in other datasets in the same situation.

We detail the weight and percentage we deduced for each field of the Kaggle Usability Score in Table 1. We report all the steps of the calculation for all the weights in the Supplementary File S1. To the best of our knowledge, ours is the first interpretation of the Kaggle Dataset Usability Score released publicly. To test the effectiveness of our interpretation of the Kaggle score, we randomly selected five datasets and utilized our weights (Table 1) to predict their published Kaggle scores: our predictions matched the published Kaggle scores in all five cases.

**Table 1:** Our interpretation of the Kaggle Dataset Usability Score. Completeness: the Kaggle Usability Score adds the indicated points if who uploaded the dataset to Kaggle included a subtitle, some tags (for example, *tabular*, *health*, *medical records*, a descriptive text on the dataset and a cover image representing the dataset. Credibilty: the Kaggle Usability Score adds the indicated points if who uploaded the dataset to Kaggle included information about the provenance of the dataset, a public notebook (an interactive computing script, made with Jupyter for example), and the information about how frequently the dataset will be updated (most of the times, *never*). Compatibility: the Kaggle Usability Score adds the indicated points if who uploaded the dataset to Kaggle included information about the license under which the dataset can be used (for example, the Creative Commons CC BY-NC 4.0 DEED license), the file format (for example, CSV, ODS, XLSX, DICOM), a text describing the file, and the explanation of the meanings of the columns, for tabular data files)

Kaggle dataset usability score			
Position	Field	Weight	Percentage
1	Subtitle	$\sim$1.17	11.7%
2	Tag	$\sim$1.17	11.7%
3	Description	$\sim$1.17	11.7%
4	Cover Image	$\sim$0.59	5.9%
	Total for Completeness	4.10	41.0%
5	Source/Provenance	$\sim$0.59	5.9%
6	Public Notebook	$\sim$0.59	5.9%
7	Update Frequency	$\sim$0.59	5.9%
	Total for Credibility	1.77	17.7%
8	License	$\sim$1.18	11.8%
9	File Format	$\sim$1.18	11.8%
10	File Description	$\sim$1.18	11.8%
11	Column Description^a^	$\sim$0.59	5.9%
	Total for Compatibility	4.13	41.3%
	Total	10.00	100.0%

^a^The Column Description field is absent for non-tabular datasets, such as datasets of medical images

The Kaggle Dataset Usability Score was not designed for scientific purposes and has some drawbacks. It weighs both trust-related aspects of a dataset (license, feature description, source and provenance) and non-scientific items, like the presence of a subtitle, tags, or cover image on the Kaggle webpage. Indeed, the non-scientific values of this score account for 41.1% of the whole score, making it unreliable for trustworthiness assessments. Our work draws inspiration from the Kaggle score refocusing the evaluation around data quality and trust.

For completeness, we noticed that the Kaggle Dataset Usability Score most of the time has eleven fields, but sometimes only ten. The *Column Description* is missing, in fact, for datasets that do not have features represented as columns. This is the case of image datasets, such as the *1980s Album Covers* dataset [71]. Some datasets have all the eleven requirements satisfied, but their final score is lower than 10.00. This is the case of the *LFW – Facial Recognition* dataset [72], for example, which has all the eleven conditions met but has 9.41 as a score, and not 10.00. A member of the Kaggle team, replying to a Kaggle user who highlighted the same problem for another dataset, called that situation an *issue* and declared it was *addressed* [68].

### Datasheets for datasets

Documentation is fundamental to improve data quality standards and support appropriate use of datasets [8, 64, 73–75]. *Datasheets for Datasets* [64] is a prominent documentation framework facilitating the communication between dataset curators and users. *Datasheets for Datasets* inform dataset users about the characteristics of a dataset, including its motivation, composition, collection, preprocessing, and intended use. They cover disparate aspects of datasets, including their size, the definition of train/test splits, relation to external resources, the presence of confidential information and offensive data. An entire set of questions focuses on dataset distribution, covering the distribution period, recipients, and regulatory restrictions such as export controls. The final set of questions targets dataset maintenance, including information on planned updates, support for older versions, and contribution mechanisms.

With their 57 questions, *Datasheets for Datasets* provide a lengthy and general-purpose blueprint to reason about data. We draw from this documentation framework, condensing it and tailoring it to biomedical data. For example, we focus on medical devices for data collection and on specific protected attributes, such as genetic ancestry, important in the medical domain. Moreover, we emphasize data governance topics that are especially salient in the EU AI Act.

*Datasheets for Datasets* was not actually designed to be automated and does not provide a coefficient or a score to assess its level in the documentation of a specific dataset: according to its authors [64], it is intended to be a series of questions, guidelines, or recommendations to elicit important information from dataset curators.

### Our proposal

In this study, we propose our Venus score to assess biomedical data quality, based on data quality provisions of the EU AI Act [62, 63, 65], drawing inspiration from core concepts of *Datasheets for Datasets* [64] and *Kaggle Dataset Usability Score* [68] The main contributions of this work are twofold: first, a set of ten questions, summarizing key requirements for data trustworthiness in the medical domain (“Method: our proposed Venus score” section), and second, the insights we gain from applying the questionnaire to twelve important biomedical datasets (“Datasets” and “Results” sections). Additionally, we provide a quantitative weighting scheme to translate questionnaire answers into numerical scores (Supplementary File S2).

## Method: our proposed Venus score

As mentioned earlier, we identified *Datasheets for Datasets* [64] and the *Kaggle Dataset Usability Score* [68] as useful frameworks for assessing data quality. The former is a wide-ranging qualitative framework to extensively document datasets. The latter is a simple quantitative approach to encourage dataset providers to follow usability guidelines.

*Datasheets for Datasets* and the *Kaggle Dataset Usability Score* suffer from some drawbacks. Even if complete and comprehensive, the list of constraints of *Datasheets for Datasets* consists of over 50 questions. We believe it is very demanding for data curators to carefully answer all 57 questions before releasing a biomedical dataset. At the same time, it is difficult for dataset users to locate key information, including data quality requirements for medical AI specified in the European Union Artificial Intelligence Act [62, 63, 65].

The *Kaggle Dataset Usability Score*, although useful, has some limitations too, as explained earlier. This coefficient gives importance both to trust-related aspects of a dataset (license, feature description, or source or provenance) and to non-scientific elements, such as the presence of subtitle, tags, or cover image for a specific dataset.

**Fig. 1:**
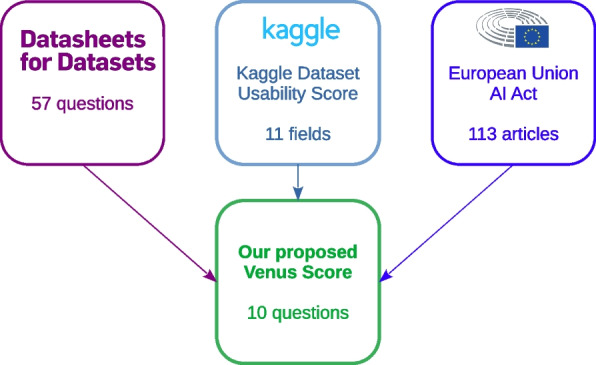
A schematic representation of the frameworks inspiring our proposed Venus score. We incorporated concepts from *Datasheets for Datasets* [64], from the Kaggle Dataset Usability Score [68], and from the European Union Artificial Intelligence Act [62] to design our proposed Venus score for assessing the quality of biomedical datasets

We overcome these limitations by proposing our Venus score for assessing the quality and the trustworthiness of biomedical data. Our framework draws its main topics from the *EU AI Act*, expanding them into concrete questions inspired by *Datasheets for Datasets*, and summarizes their fulfillment with a quantitative score inspired by the *Kaggle Dataset Usability Score*.

We define *data trustworthiness* in the medical domain as the ability of data and accompanying documentation to lawfully support reliable models and analyses, which are cognizant of their own validity boundaries, potential for generalization, limitations, and plausible blind spots [76].

The Venus score, detailed in this section, collates the data quality requirements from the *EU AI Act* [62] (Fig. 1), the key aspects from *Datasheets for Datasets* [64], and the condensed core of the *Kaggle Dataset Usability Score* [67].

Here we present the ten questions of the novel Venus score to measure data quality and trustworthiness. A biomedical dataset undergoing this evaluation gets a real value between 0.00 and 1.00 for each of the ten questions, consequently having 0.00 as a minimum and 10.00 as a maximum possible score. We describe the main subtopics for each question highlighting them in *italics*. Overall, the Venus score captures data quality and trustworthiness favoring informed adoption of datasets and compliance with the EU AI Act. Similarly to *Datasheets for Datasets* [64], the calculation of the Venus score is not intended to be automated: it should be manually assessed. For simplicity, we also provide the Venus score checklist and weighting scheme as a stand-alone, fillable spreadsheet (Supplementary File S2). Dataset evaluators can answer the sub-questions of our ten guidelines and assign their corresponding partial sub-scores based on the information regarding that specific sub-question they find in the dataset’s documentation or scientific article.

Our Venus score can be seen simultaneously as a numerical coefficient, as a series of recommendations for data quality, as a set of guidelines, as a method or a framework for data quality assessment: each reader can decide to use it according to their needs.

### Q1: Are the origin, context, and purpose of the dataset defined?

This question supports contextual reasoning about the data. A positive answer means that documentation informs prospective dataset users as to who curated the data (*curators*) and why it was collected (*purpose*). Additionally, it should provide information on the setting, data subjects, and collection methods (*context*). For example, documentation should clarify whether the data was collected during patient care, clinical, or non-clinical trials. This information influences expectations for answers to subsequent questions, including best practices for data protection.

The Arrhythmia dataset is a negative example for this question as it provides no information on its provenance and context [77]. CheXpert [78], on the other hand, is well documented in accompanying literature describing its purpose and curation strategy [79]. This question supports reasoning about the true potential of AI models trained on this dataset, including their generalization to clinical settings [80].

### Q2: Are data protection measures described? Is there a license for the data?

Data curators and collectors should take measures to protect the patients’ data [81, 82]. Data documentation should describe whether and how patients consented to data collection and secondary use (*consent*) and which steps were taken to avoid the released data being linked back to individual patients (*deidentification*). For instance, curators can describe protected health information (PHI) and its removal. *Terms of use* and *data licenses* may further protect data by specifying acceptable use, including best practices for data management and agreements to not attempt identifying individuals in the dataset.

For example, most of the datasets publicly available in the University of California Irvine Machine Learning Repository [31] are released under the open Creative Commons Attribution 4.0 International (CC BY 4.0) license [83], and anyone can download them without restrictions. Conversely, the intensive care unit MIMIC-IV dataset [48, 84] was licensed under the PhysioNet Credentialed Health Data License 1.5.0. To download it, users first need to attend the MIT Collaborative Institutional Training Initiative (CITI) required training on *Data or Specimens Only Research* and then sign the data use agreement on PhysioNet [84, 85].

### Q3: Are the devices, medical centers, and collection periods clearly identified?

This question regards how, when, and where the data entries of the dataset were collected. Dataset curators should provide information on these three aspects. Information on the devices should include the type of biomedical machinery that collected the data and, if possible, its model and brand (*devices*). This information is particularly important in the bioinformatics context, where a plethora of platforms to collect gene expression data from microarray, bulk RNA-seq, and single-cell RNA-seq exist, which are often incompatible with each other and need batch correction. Additionally, documentation should provide as much context as possible as to where the data was collected (*medical centers*) and over which time frame (*collection period*). This helps users estimate how recent and representative the data is for their application.

For example, the webpage on Gene Expression Omnibus (GEO) containing information about the GSE89413 single-cell RNA-seq dataset released by Jo Lynne Harenza and colleagues [86] includes details about the RNA-seq platform employed to collect the data: GPL18573 Illumina NextSeq 500 (Homo sapiens) [87]. Insights about when and where the data were collected are pivotal, too. For example, Guillaume Le Gall and colleagues [88] released a dataset of electronic health records of patients diagnosed with ischemic heart disease, cerebrovascular arterial disease, and with inflammatory bowel disease on Figshare in 2017 [89], specifying, in the associated article, that data were gathered at Hôpital Saint-Antoine (Paris, France, EU) between 1996 and 2015 [88].

### Q4: Are all variables and their values properly explained?

To get a full point on this requirement, the documentation of the dataset should provide an explanation of the meaning of each variable (*features* – encoded as columns in tabular datasets) and the meanings they may have (*values*). The Kaggle Dataset Usability Score follows the same guideline regarding the features, where it requires a *column description*: as obvious as it sounds, all variables should be documented. Unfortunately, this is not the case in several datasets which lack this information [90, 91].

The description of the values of the features should be included, too. If the “sex” variable, for example, can have values 0 or 1, the data curators should specify if 0 or 1 means “man” or “woman”. Similarly, if the variable named “XYZ” ranges in the $[-2.532; +10.701]$ real interval, the dataset authors should explain what “XYZ” stands for and what is the meaning of the values in that specific interval.

These recommendations might sound trivial, but there are plenty of datasets online whose features are undocumented and whose values are unexplained.

### Q5: Does the dataset documentation include information on subpopulations identified by protected attributes such as the age, sex, ethnicity, and genetic ancestry of patients?

This question encourages reasoning about inclusion, bias, and anti-discrimination. Dataset users should know the marginal distribution of sensitive attributes, to detect the under-representation of specific subpopulations (*marginal distribution*). Thorough documentation summarizes the joint distribution between demographic attributes and important variables (*joint distribution*), including target variables for supervised machine learning problems. Complete documentation should also describe expected patterns in the marginal and joint distribution, motivating them with supporting evidence, such as studies describing the correlation between age and diabetes (*expected distribution*) [92]. Documentation should report how demographic information was obtained, describing, for example, whether labels were self-reported by patients or annotated externally (*label provenance*) [15].

### Q6: Are sources of potential inaccuracy listed and characterized?

Data accuracy is a fundamental requirement, also enshrined by data protection regulation [93]. Curators should inform dataset users by listing the most plausible sources of data inaccuracy (*listed sources*). For instance, errors at different stages can affect biomedical data as it goes from human subjects to medical records, to databases, to dataset curators, and to dataset users [94]. Patients can report inaccurate data about themselves, including medication dosage. Medical personnel can introduce clerical errors in EHRs by mistyping, inverting digits, or selecting the wrong option in a dropdown menu. Fragmentation may lead to incomplete medical records [95]. Ideally, curators should also quantify the severity of these inaccuracies (*characterized*). This is especially important for labels automatically annotated by machine learning models, including medical conditions extracted from reports written in human language [79]. Errors sizable enough to affect researchers’ conclusions have a large potential to harm the reliability of findings, the trustworthiness of models, and the quality of new healthcare standards [96]. Quantitative and qualitative descriptions of different data inaccuracies and their severity are invaluable for dataset users [79, 97, 98].

### Q7: Is the information about noise included?

Noise is an undesired part of the biomedical signal that is unintentionally recorded by the machinery and might make it more difficult to recognize the desirable part of the signal. Since most biomedical data come with noise, curators should always list the most plausible sources of noise (*listed sources*). Medical images come with Gaussian noise due to electronic interference, sensor limitations, or image processing techniques [99]. Microarray and RNA-seq gene expression come with noise signals called *batch effects* [100, 101], due to the different conditions of the experiments carried out to generate the data themselves. Electrocardiogram (ECG) heart data come with noise called *baseline wander* that can be caused by the respiration or the motion of the patients [102]. Electroencephalography (EEG) brain signals, in particular, come with several different noise sources and artifacts that can be due to the movements of the patients, such as jaw clenching, eye movements, electrical interference, and environmental factors [103]. Cheminformatics data often need noise cleaning, as well [104]. Of course, before any computational analysis, noise should be removed or mitigated to the extent possible.

Noise is a tricky aspect of biomedical informatics research: since it is invisible to beginners and researchers who do not know the data well, it might be overlooked, producing wrong or misleading results. Therefore, we recommend including information about the noise of the dataset in the dataset documentation, so that dataset users can take care of it effectively. Awareness is the first step. Documentation should also inform dataset users about the noise magnitude (*characterized*). Indeed, it is especially important for practitioners and researchers to understand what signal-to-noise ratio [105] is required to independently reproduce equivalent setups for their data in different geographical, contextual, or functional settings.

### Q8: Does the documentation describe data preparation, including cleaning and annotation?

Data is typically preprocessed to reduce inaccuracies and augment the available information. Inaccuracy reduction includes signal filtering, data imputation, and outlier removal [106] (*cleaning*). These interventions can have a sizable impact on dataset representativeness and inclusivity. Moreover, curators often augment biomedical data with additional labels, for example indicating the skin type of a patient [107] (*annotation*). We reiterate that labels can be ambiguous and subjective; labeling procedures should be described as precisely as possible, including who is involved and how the annotation is performed. Finally, before publishing a dataset, entries are often protected by altering variables that support identification, such as hospitalization dates [47].

Transparent documentation of data preprocessing supports informed dataset usage. For example, researchers can gauge the reliability of automatically generated labels and the extent to which they can be regarded as ground truth [79]. Practitioners at medical centers can better align their data processing pipelines to replicate the curators’ setting. Overall, dataset users have more agency over data if they understand the processes and choices that shape it [15].

### Q9: Is there a peer-reviewed scientific publication describing the data?

If a released dataset is accompanied by a scientific publication in a reputable journal, we consider it worthy of an extra point in our Venus score. The article should focus on the dataset and describe it in as much detail as possible (*data-centric publication*). A scientific paper that underwent independent peer-review and was then published in a biomedical journal indexed for example in PubMed [108] can provide additional guarantees for a dataset. This represents a form of permanent documentation guaranteeing data transparency regardless of future updates to data repositories. Not all publications are equal: as a rule of thumb, we recommend prioritizing open-access journals indexed in the Q1 category for health informatics or molecular biology of ScimagoJR [109, 110] (*reputable*).

Several reliable datasets were released together with trustworthy scientific publications, such as the MIMIC-III [48] dataset of electronic health records and the GSE89413 [86] dataset of single-cell RNA-seq. A scientific article about a dataset contains relevant information about it and favours the permanence of this information online: each paper published in an indexed scientific journal, in fact, has a unique code (called digital object identifier – DOI) and a public URL, that will be available online for a long time. In the case of open-access journals, the articles will be publicly available for free to anyone.

On the contrary, datasets without a publication and that only have a descriptive webpage on Kaggle or on the UC Irvine ML Repository, typically have a more volatile documentation: those webpages can be deleted or edited at any time.

### Q10: Is the dataset available online without restrictions and does it have a global ID associated?

The last question of our score regards the public availability of the dataset and whether a stable global persistent web identifier has been associated with it. Of course, publicly available datasets are the pillar of open science, in biomedical informatics, too. Datasets that are openly available without restrictions on the internet can be analyzed by anyone, at any time, and by any means, thereby encouraging more scrutiny and accelerating scientific progress [111] (*unrestricted*).

Biomedical datasets can be publicly released on several websites, such as Figshare [29], Zenodo [30], and University of California Irvine Machine Learning Repository [31]. These websites, in addition to keeping datasets openly available to anyone, also associate a digital object identifier (DOI) with each of them, making them unique, findable, and durable (*persistent*). Datasets on these websites can be browsed and found through data search engines such as re3data [36], or Google Dataset Search [37]. Since retracted datasets can remain available through third-party sites such as academic torrents [112, 113], we stress the importance of obtaining datasets from an official release made by its curators (*official*).

### Overlap with EU AI Act, *Datasheets for Dataset* and *Kaggle Dataset Usability Score*

To be more precise regarding the relationship with the EU AI Act, the *Kaggle Dataset Usability Score*, and *Datasheets for Datasets*, we report in Table 2 the mapping of each Venus score question with the fields and requirements of these three guidelines. The Venus score questions cover the AI Act data governance requirements and include two additional requisites on transparency related to open data and documentation. The Venus score has minor overlaps with the *Kaggle Dataset Usability Score*, which covers data provenance, licensing, and variable description. The remaining fields from the *Kaggle Dataset Usability Score* are clearly unrelated to it.

**Table 2:** Main frameworks informing the Venus score. Questions from the Venus score support specific requirements from the AI Act as well as additional requirements for the availability of data and documentation. Each question is mapped to corresponding Datasheet items and Kaggle fields

Venus score	EU AI act requirements questions	Datasheets for datasets questions	Kaggle score fields	Venus score additional desiderata
Q1	origin of data; purpose of data collection	1, 2, 21	provenance	
Q2	privacy & data protection	15, 18, 28, 29, 46	license	
Q3	geographic, contextual, & functional setting	22, 25		
Q4	information represented by the data	9	column	
			description	
Q5	possible biases	17		
Q6	errors & shortcomings	10, 13		
Q7	errors & shortcomings	13		
Q8	data preparation	33, 40		
Q9				perm. doc. &
				ext. val.
Q10		44, 45, 46		open data

P*erm. doc *Permanent documentation, *Ext. val *External validation, *Kaggle score fields *fields of the Kaggle Dataset Usability Score

The third column of Table 2 lists the *Datasheets for Datasets* questions related to the EU AI act requirements and the additional desiderata covered by the Venus score. The frameworks differ in several ways. First, our questions are focused on the EU AI Act and the biomedical domain. For example, the 17th question of *Datasheets for Datasets* mentions subpopulations and their distribution and is therefore *generally* related to possible biases. Q5 in the Venus score recognizes the importance of joint distributions between target and sensitive attributes for algorithmic discrimination [114] and identifies attributes that are especially salient and conducive to inequitable outcomes in the medical domain [115]. Second, over 50% of the *Datasheets fo Datasets* questions are not relevant to the EU data requirements and, therefore, unrelated to our proposed framework. For example, there is no overlap with the 12th question (“Are there recommended data splits (for example, training, development/validation, testing)?”) since it is specific to datasets used as public benchmarks, or with the 14th question (“Is the dataset self-contained?”), which is always true in our case.

Additionally, there is no overlap with the 37th–41st questions on data use or the 50th–57th questions on data maintenance, because the EU AI act does not make specific provisions on these aspects.

Moreover, the 4th, 20th, 32nd, 36th, 49th, and 57th questions of *Datasheets for Datasets*, all asking the same question (“Any other comments?”) did not find room in our Venus score recommendations because of their ambiguity. All in all, only 20 questions out of 57 of *Datasheets for Datasets* are covered by our Venus score guidelines, that is 35%, clearly highlighting the different nature of our proposed set of questions.

## Datasets

To test the effectiveness of our Venus score, we selected and collected twelve different biomedical datasets from different fields (health informatics, bioinformatics, physiological signal processing, digital pathology, cheminformatics, and medical text), with different diseases (cancer, cardiac diseases, neurological illnesses, colon disease), available on multiple platforms (UC Irvine ML Repo, Figshare, PhysioNet, CRAN, GEO, ArrayExpress, TCIA, DREAM Challenges, and GitHub), and with different levels of data access restrictions (Table 3).

### Electronic health records

Four datasets contain data from electronic health records, covering different diseases and available on different platforms: Mesothelioma Turkey [90], IBD Paris [88, 89], MIMIC-III [47, 84, 116], Paquid [117, 118].

Among them, MIMIC-III is perhaps the most famous dataset of intensive care unit (ICU) available online and, as the name suggests, it is the third release from a database of patients admitted to the critical care units of the Beth Israel Deaconess Medical Center (USA): its predecessors were MIMIC [119] and MIMIC-II [120], and its only successor for the moment is MIMIC-IV [48]. MIMIC-III is available on the curated PhysioNet [46, 121] platform, with some restrictions, and it was released with a publication in the Scientific Data journal [47].

The Mesothelioma Turkey dataset [90, 91] contains data from 324 Turkish patients with lung cancer and was released on the UC Irvine (UCI) ML Repository in 2016. Like all datasets on the UCI ML Repo, it can be downloaded easily without any restriction or registration as a ZIP file with a single click.

Paquid is the only dataset of this list available within a software package – the lcmm software library in R [117, 122] – and contains longitudinal data of patients with mental health conditions. It can be downloaded by installing the lcmm package within an R environment, without restrictions, although a basic familiarity with R is required. The IBD Paris dataset [88, 89], instead, was released by its curators on Figshare together with its peer-reviewed publication, and contains medical records of 90 French patients.

### Bioinformatics

In this domain, we selected three datasets covering two diseases and two different platforms: GSE16476 [123, 124] from GEO [40], E-MTAB-8248 [125, 126] from ArrayExpress [41], and GSE79209 [127, 128] also from GEO. The former two contain microarray gene expression data, while the latter contains the more modern and advanced bulk RNA-seq data. These datasets are available on the just-mentioned platforms for download without restrictions.

### Physiologic signal processing

We selected two datasets within this important field of biomedical informatics from PhysioNet. Both contain electrogram data: the EEG Motor Movement/Imagery [129, 130] of electroencephalography brain data and the MIT-BIH ECG Arrhythmia dataset [131–133] of electrocardiogram heart data. The Motor Movement/Imagery dataset contains 1,500 EEG recordings of 109 subjects with several neurological conditions, who performed experiments involving body movements. The MIT-BIH dataset consists of ECG data from 49 patients with cardiac arrhythmias, which are irregular beats of the heart.

### Medical imaging

From medical imaging, we included the Breast Cancer Screening Digital Breast Tomosynthesis (DBT) dataset [134, 135], openly available on the Cancer Imaging Archive [44]. This dataset consists of DICOM files and, compared to the other datasets, stands out for its huge memory occupation: images in the training set occupy 1.42 terabytes (TB). In comparison, the IBD Paris [88] dataset mentioned earlier consists of a single XLSX file of 12.98 kilobytes (kB). The training set of the Breast Cancer Screening DBT occupies 110 million times as much space. The Breast Cancer Screening DBT dataset can be freely downloaded from TCIA without restrictions and without registration.

### Chemical informatics

We also evaluate the DREAM Drug Synergy Challenge [136, 137] dataset, a cheminformatics data resource publicly available on Synapse.org. A DREAM challenge is an online free competition where organizers provide data, data description, scientific questions to answer through data science tools, and a platform to upload results. Anyone can register for the challenge, download the data, analyze them, and submit their predictions on the test set. Teams that provide results closer to the test set ground truth win the challenge, and their members get the chance to be listed as first authors in the scientific paper written about the challenge. This dataset contains pharmacology and molecular data provided by the AstraZeneca and Sanger pharma companies with the goal of predicting chemical compounds of effective drug combinations for cancer via machine learning. The dataset is available for users registered to the challenge on Synapse.org.

### Medical text

The last dataset we utilized belongs to the medical text domain: MedQuad [138, 139]. MedQuad contains the text of 47,457 medical question-answer pairs derived from several websites, together with annotated XML files to facilitate information retrieval and NLP tasks. The dataset was originally released on GitHub, where it is still available without restrictions, but is also present on Kaggle and Hugging Face.

### Annotation process

We collected information on each dataset from two sources, namely the main repositories where datasets have been published and the accompanying peer-reviewed publications, if any. If a dataset was available in multiple repositories, we selected the original release when unambiguous; otherwise, we chose the repository linked by DataMed [140]. Within a dataset repository, we collected information concerning that dataset from all its subpages (for example, “About”, “Getting started”, “Release notes”, ...) and consulted the terms of use and default licensing from the repository. From publications, we carefully read all sections, including supplementary materials. We consulted the publication referenced in the landing page of a dataset; if more than one publication was referenced, we focused on the main (first) one. Based on this information, two authors annotated the Venus scores independently. We then worked on resolving disagreements, going from an initially strong (Pearson’s correlation coefficient $\rho =0.66$) to a very strong final inter-annotator agreement($\rho =0.96$), as measured by Pearson’s correlation coefficient [141–143]; we finally averaged the annotations. We report additional details in Table 3, including the references we consulted for each dataset.

## Results

We manually applied the ten questions of our Venus score described in “Method: our proposed Venus score” section to the twelve datasets outlined in “Datasets” section, and we reported all the detailed results in Table 4. This section presents our results. First, we report a detailed analysis of data quality for each dataset (detailed analysis). Then, we describe the main trends emerging from this analysis (main trends). We summarized the numerical results in Table 4.

**Table 3:** Schematic description of the datasets. ^a^critical care: sepsis, acute respiratory distress syndrome, heart failure, pneumonia, and others. ^b^multiple cancers: primarily colon cancer, lung cancer, and breast cancer. References: reference to the article provided by the dataset landing webpage

**ID name**	**field**	**data type**	
D1 Mesothelioma Turkey	medical informatics	EHRs	
D2 IBD Paris	medical informatics	EHRs	
D3 MIMIC-III	medical informatics	EHRs	
D4 Paquid	medical informatics	EHRs	
D5 GSE16476	bioinformatics	microarray gene expression	
D6 E-MTAB-8248	bioinformatics	microarray gene expression	
D7 GSE79209	bioinformatics	bulk RNA-seq gene expression	
D8 EEG Motor Movement/Imagery	physiologic signals	brain EEG	
D9 MIT-BIH ECG Arrhythmia	physiologic signals	heart ECG	
D10 Breast Cancer Screening DBT	digital pathology	medical images	
D11 DREAM Drug Synergy Challenge	cheminformatics	pharmacology and molecular data	
D12 MedQuad	NLP	text	
**ID name**	**disease**	**repository**	**references**
D1 Mesothelioma Turkey	mesothelioma	UC Irvine ML Repo	[90]
D2 IBD Paris	IBD, IHD, and CVAD	Figshare	[88, 89]
D3 MIMIC-III	critical care^a^	PhysioNet	[47, 116]
D4 Paquid	dementia	CRAN	[117, 118]
D5 GSE16476	neuroblastoma	GEO	[123, 124]
D6 E-MTAB-8248	neuroblastoma	ArrayExpress	[125, 126]
D7 GSE79209	lung cancer	GEO	[127, 128]
D8 EEG Motor Movement/Imagery	neurologic conditions	PhysioNet	[129, 130]
D9 MIT-BIH ECG Arrhythmia	arrhythmia	PhysioNet	[131, 132]
D10 Breast Cancer Screening DBT	breast cancer	TCIA	[134, 135]
D11 DREAM Drug Synergy Challenge	multiple cancers^b^	DREAM Challenges / Synapse	[136, 137]
D12 MedQuad	multiple diseases	GitHub	[138, 139]

*BIT *Beth Israel Hospital, *CRAN *Comprehensive R Archive Network, *CVAD *Cerebrovascular arterial disease, *DBT *Digital Breast Tomosynthesis, *DREAM *Dialogue for Reverse Engineering Assessment and Methods, *ECG *Electrocardiogram, electrocardiography, *EEG *Electroencephalography, *EHRs *Electronic health records, *GEO *Gene Expression Omnibus, *IBD *Inflammatory bowel disease, *IHD *Ischemic heart disease, *MIMIC *Medical Information Mart for Intensive Care, *NLP *Natural language processing, *RNA-seq *RNA-sequencing, *TCIA *The Cancer Imaging Archive, *UC Irvine ML Repo *University of California Irvine Machine Learning Repository

**Table 4:** Results of the application of our Venus score to the twelve datasets. The description of the Q1–Q10 questions of our Venus score can be found in “Method: our proposed Venus score” section, and the description of the D1–D12 datasets can be found in “Datasets” section

	**D1**	**D2**	**D3**	**D4**
	**Mesoth. Turkey**	**IBD Paris**	**MIMIC-III**	**Paquid**
Q1 Origin, context, and purpose	0.3	1	1	0.65
Q2 Data protection measures and license	0.35	0.6	1	0
Q3 Devices, medical centers, & collection periods	0.15	0.75	1	0.7
Q4 All variables and their values explained	0.4	0.85	1	0.9
Q5 Protected attributes	0.5	0.63	0.5	0.95
Q6 Potential inaccuracy	0	0	0.3	0.3
Q7 Noise	0	0	0.05	0
Q8 Data preparation	0	0.65	0.6	0.32
Q9 Peer-reviewed scientific publication	0	1	1	0.9
Q10 Online open availability and global ID	1	1	1	0.3
total	2.7	6.48	7.45	5.02
	**D5**	**D6**	**D7**	**D8**
	**GSE16476**	**E-MTAB-8248**	**GSE79209**	**EEG Motor**
Q1 Origin, context, and purpose	0.8	1	1	0.6
Q2 Data protection measures and license	0.15	0.45	0.7	0.25
Q3 Devices, medical centers, & collection periods	0.6	0.5	1	0.35
Q4 All variables and their values explained	1	1	1	0.6
Q5 Protected attributes	0.7	0.5	0.85	0
Q6 Potential inaccuracy	0	0	0.2	0
Q7 Noise	0.05	0.15	0.35	0
Q8 Data preparation	0.42	0.32	1	0.4
Q9 Peer-reviewed scientific publication	1	0.5	1	0.5
Q10 Online open availability and global ID	1	1	1	1
total	5.72	5.42	8.1	3.7
	**D9**	**D10**	**D11**	**D12**
	**MIT-BIH ECG**	**Breast DBT**	**DREAM drugs**	**MedQuad**
Q1 Origin, context, and purpose	1	1	0.9	1
Q2 Data protection measures and license	0.25	0.5	0.45	0.45
Q3 Devices, medical centers, & collection periods	1	0.6	0.75	0.6
Q4 All variables and their values explained	0.42	1	1	1
Q5 Protected attributes	0.4	0.5	0.05	0
Q6 Potential inaccuracy	0.9	0.1	0.3	0.1
Q7 Noise	1	0	0.2	0.1
Q8 Data preparation	1	0.9	0.58	0.58
Q9 Peer-reviewed scientific publication	1	1	0.75	1
Q10 Online open availability and global ID	1	1	0.75	0.6
total	7.97	6.6	5.73	5.43
	**average for**	**stdev for**	**min for**	**max for**
	**each**	**each**	**each**	**each**
	**question**	**question**	**question**	**question**
Q1 Origin, context, and purpose	0.85	0.23	0.3	1
Q2 Data protection measures and license	0.43	0.23	0.3	1
Q3 Devices, medical centers, & collection periods	0.67	0.23	0.15	1
Q4 All variables and their values explained	0.85	0.26	0.15	1
Q5 Protected attributes	0.46	0.26	0	0.95
Q6 Potential inaccuracy	0.18	0.31	0	0.9
Q7 Noise	0.16	0.31	0	1
Q8 Data preparation	0.56	0.29	0	1
Q9 Peer-reviewed scientific publication	0.8	0.29	0	1
Q10 Online open availability and global ID	0.89	0.32	0	1
total	5.86	0.32	2.7	8.1

*DBT* Digital Breast Tomosynthesis, *DREAM *Dialogue for Reverse Engineering Assessment and Methods, *ECG* Electrocardiogram, electrocardiography, *EEG *Electroencephalography, *IBD *Inflammatory bowel disease, *MIT-BIH *Massachusetts Institute of Technology-Beth Israel Hospital, *Mesoth *Mesothelioma, *stdev *standard deviation

### Detailed analysis

For each question and each dataset, we gave a score of 0 if the specified information is completely absent from the dataset documentation (article and webpage) or, vice versa, 1 if all aspects of a question are suitably treated. When partial information was present, we inserted a real number in the [0.1; 0.9] interval.

The D1 dataset on Turkish mesothelioma has no information on potential inaccuracy (Q6), noise (Q7), and data preparation (Q8). On the other hand, it meets all the criteria for public availability (Q10). Moreover, D1 partially satisfies the first five requirements of the Venus score: we noticed some information on demographic attributes (Q5) and saw that the variables are documented, but not their values (Q4). Basic information on the context where the data were collected and on the purpose of this dataset are also lacking (Q1), making D1 the lowest-scoring dataset for this question. We assigned a final mark of 2.70 out of 10 for this dataset, which is the lowest among the twelve datasets analyzed (Table 4).

The D2 French inflammatory bowel disease dataset is fully available on Figshare [89] (Q10), with an accompanying peer-reviewed publication [88] (Q9), which provides complete contextual information (Q1). We assigned partial, high marks to the questions on data protection (Q2), devices, medical centers and collection periods (Q3), finding most of the information in the dataset article. The majority of the variables and their values are correctly documented (Q4), and the marginal distribution of sex and age is characterized (Q5). We found no information on the potential inaccuracy and the noise within the dataset (Q6 and Q7). All in all, we assigned 6.48 out of 10 to this dataset.

The third dataset we assessed is D3 MIMIC-III, which obtained a high mark of 7.45. Six questions had complete information and therefore maximum points (Q1–Q4, Q9, Q10). We found just a mention regarding the device name, and little information on sources of inaccuracy (Q6) and data cleaning (Q8) in the dataset release notes. A wide array of protected attributes are available with the data (Q5), including patients’ insurance status, language, and religion, allowing for the computation of rich marginal and joint statistics.

For Paquid (D4), we found no information regarding data protection measures (Q2), and noise (Q7). The dataset is only available within a software library of R and without a unique identifier (Q10). Collection periods are available (Q3), however, we found no information on how the subset of the original cohort was selected or how these data were collected. Partial and insufficient information about potential inaccuracy (Q6) and data preparation (Q8) was identified in the dataset documentation. Paquid identifies subpopulations based on educational level, gender, and age; the accompanying documentation describes their joint distribution with Alzheimer’s disease and dementia, providing reference values from the literature (Q5). The overall score is 5.02.

Moving on to gene expression data, the D5 GSE16476 dataset, describing patients diagnosed with neuroblastoma, represents a mixed bag. On the positive side, we found all the information regarding the variables (gene probesets [144] within the specified Affymetrix platform) and their values (Q4), a peer-reviewed publication (Q9), and the open availability of the dataset with a stable ID on GEO (Q10). Critically, we found little information on data protection (Q2), data curation purpose (Q3), sources of inaccuracy (Q6), and noise (Q7). This resulted in a score of 5.72; overall, the dataset seems to be released more for experts who already have a deep knowledge of microarray gene expression and Affymetrix than for beginners.

For D6 (E-MTAB-8248), also covering neuroblastoma and microarray gene expression, we found complete information regarding origin, context, and purpose (Q1), the documentation for all the variables and their values (Q5), and we noticed open availability for the dataset without restrictions and including a DOI on ArrayExpress (Q10). We found no information on collection periods (Q3) and limited information on data protection (Q2); the accompanying peer-reviewed publication focuses on analyzing the data rather than presenting it (Q9), limiting its utility. We found no information about sources of inaccuracy (Q6) and noise (Q7). We therefore assigned to this dataset a mark of 5.42 out of 10.

The D7 GSE79209 dataset stands out as highly trustworthy with an overall score of 8.1. Six questions of the Venus score (Q1, Q3, Q4, Q8, Q9, and Q10) are completely satisfied, and two deserved partial, high scores: curators paid attention to data protection (Q2), mentioning the informed consent elicitation, and to protected attributes such as sex and age, including their joint distribution with pre-malignant lesion status (Q5). This dataset attained low scores only for potential inaccuracy (Q6) and noise (Q7), which are not sufficiently described. Overall, this dataset release represents a great example of trustworthy data thanks to its thorough documentation.

The D8 EEG Motor Movement/Imagery dataset, conversely, has several drawbacks. We noticed no information on demographic attributes (Q5), potential inaccuracy (Q6), and noise (Q7); we found information about the dataset license but not on data protection (Q2). Medical centers and collection periods are not mentioned (Q3) and the description of variables and their values is incomplete (Q4). Moreover, we noticed that the scientific article linked to this dataset [130] is about the technology used to collect this dataset rather than the data themselves. We eventually assigned a score of 3.7 to this dataset which, even if available on the popular platform PhysioNet, lacks pivotal information.

The D9 MIT-BIH ECG dataset, also consisting of electrogram data stored on PhysioNet, obtained the second-best mark of our study: 7.97 out of 10. This dataset attained maximum scores for 6 questions (Q1, Q3, Q7, Q8, Q9, and Q10), and a high score for the information on potential inaccuracy (Q6), which was clearly explained and characterized in the article, including, for instance, the frequency of the main disturbances. The documentation is lacking in regards to data protection (Q2), variable explanation (Q4), and protected subpopulations (Q5).

The D10 Breast Cancer DBT provides thorough information on its variables (Q4), origin and context (Q1) through a data-centric peer-reviewed publication (Q9) and an open data release (Q10); data preparation is also described in detail (Q8). On the other hand, we found almost no information about noise (Q7), potential inaccuracy (Q6), and medical devices (Q3), and partial information on data protection (Q2) and protected attributes (Q5). In the end, we assigned a mark of 6.6 out of 10 to this dataset.

The D11 DREAM Drug Synergy Challenge dataset, focusing on cheminformatics, obtained an overall score of 5.73. This dataset obtained no score equal to 0 and only one equal to 1, for the variables’ explanations and their documentation (Q4). The dataset fares well in questions on origin, context, and purpose (Q1), devices, medical centers, and collection periods (Q3), but lacks detail in other scientific and technical questions. The accompanying peer-reviewed publication [137] focuses on the results of the DREAM challenge rather than the dataset itself (Q9). The dataset is available after registration on Synapse.org (Q10).

D12 MedQuad also provides mixed results with high scores for context (Q1) and peer-reviewed publication (Q9), intermediate scores for data protection (Q2) and collection period (Q3), and low scores for protected attributes (Q5) and sources of inaccuracy (Q6). It is worth noting that MedQuad is publicly available, but does not have a DOI (Q10), making it difficult to unambiguously and persistently reference it. In the end, we assigned the 5.43 mark to this dataset (Table 4).

### Main trends

The summary statistics in the bottom part of Table 4 show that most datasets have high scores for origin, context and purpose (Q1 average = 0.85), explanation of variables and their values (Q4 average = 0.85), presence of a peer-reviewed publication (Q9 average = 0.8), and public availability (Q10 average = 0.89). High scores for Q9 and Q10 come with no surprise, since we selected mostly public datasets and it is common practice for curators of biomedical datasets to release a peer-reviewed publication about them. Also in line with curators’ ambition to increase the adoption of their datasets is providing enough information about the context surrounding a dataset (Q1) and its variables (Q4). On the other hand, the twelve datasets attained low scores for sources of potential inaccuracy (Q6 average = 0.18) and noise (Q7 average = 0.16): unfortunately, these two important aspects are often neglected in dataset documentation [145]. We analyze and interpret this finding in more detail in “Discussion and conclusions” section.

Among questions with more nuanced results, we find that data protection measures can be neglected (Q2 average = 0.43). Most datasets specify a license and, less frequently, stringent terms of use that explicitly forbid the identification of individuals (MIMIC-III). Very little information is provided on consent and de-identification. For de-identification, we found only one description of the adopted measures (MIMIC-III). For consent, two datasets mention consent waivers (Breast DBT, and MIMIC-III), and two datasets say that individual consent was obtained (Paquid, and GSE79209), without describing the elicitation procedure. Finally, three datasets mention IRB approval from faculty or data protection authorities (IBD Paris, E-MTAB-8248, and GSE79209). Biomedical data exist in a complex privacy landscape with evolving best practices [146, 147]. The surveyed resources give users little information to decide whether they can ethically and lawfully process this data.

Some protected attributes are available with most datasets (Q5 average = 0.46). Typically they encode information on sex and age. Less common attributes include insurance (MIMIC-III), marital status (MIMIC-III), religion (MIMIC-III), language (MIMIC-III), ethnicity (MIMIC-III), race (Breast DBT), and education (Paquid). Documentation does not report how this data was obtained, despite the importance of this information [148]. Additionally, it is exceedingly rare for documentation to describe the joint distribution of demographic attributes and important variables in the dataset. Paquid is an exception; accompanying documentation provides a thorough discussion on the incidence of Alzheimer’s disease and dementia across age and sex [118].

The highest-scoring datasets are GSE79209 (average = 8.1), MIT-BIH ECG Arrhythmia (average = 7.97), and MIMIC-III (average = 7.45). They are hosted in specialized repositories such Gene Expression Omnibus and PhysioNet with well-specified domain-specific documentation requirements [149, 150]. The lowest-scoring datasets are Mesothelioma Turkey (average = 2.7) and EEG Motor Movement/Imagery (average = 3.7). The former is released on the UC Irvine ML Repo, a general-purpose machine learning repository. The latter was published on PhysioNet in 2009, ten years before the platform released its detailed guidelines for data sharing.

## Discussion and conclusions

### Discussion

A growing number of biomedical datasets have become available online in the last decades, supporting the development of numerous data science projects and AI applications worldwide. Secondary usage of biomedical data, in particular, can facilitate new scientific discoveries with a strong impact on patients. A statement on the website of the American Medical Informatics Association (AMIA) on the importance of secondary data analysis in biomedical sciences, in 2008, asserts:

“Secondary use of health data can enhance health care experiences for individuals, expand knowledge about disease and appropriate treatments, strengthen understanding about the effectiveness and efficiency of our health care systems, support public health and security goals, and aid businesses in meeting the needs of their customers.” [22, 151]

Unfortunately, not all datasets are of sufficient quality and trustworthiness to be utilized in a biomedical study: some of them have errors, inconsistencies, or drawbacks that make them unreliable. Sometimes, these problems are not immediately evident and, therefore, can be overlooked or neglected by data science practitioners, obtaining misleading results. These misleading results, in turn, if taken into account by medical doctors, can produce negative consequences on patients [11]. The main principle of medicine is *primum, non nocere* (first, do no harm): we firmly believe this rule is also valid for data science and computational intelligence applied to biomedical research [152].

We developed our Venus score and its questions with this goal in mind: to provide a tool that can assess the reliability and trustworthiness of biomedical data. A high Venus score denotes a low barrier for appropriate data usage and indicates the availability of suitable information to compile technical documentation, in line with data governance requirements set forth in recent regulation [62]. Overall it supports informed choices for dataset users and encourages dataset curators to share appropriate information. It is worth noting that the Venus score does not apply only to online datasets, but also to datasets received *in person* from medical professionals at a hospital, for example. If the quality and the trustworthiness of a biomedical dataset are insufficient, we advise researchers to demand the missing information or to seek alternative data of higher quality.

Applying the Venus score to popular datasets we surfaced a worrying trend, namely a generalized lack of information on noise and potential sources of inaccuracy in biomedical datasets. This information is crucial to let practitioners and researchers reason about the results they obtain from the data and how they may generalize to new settings. Unfortunately, so far, dataset curators have had few incentives to reflect on the limitations and undesirable aspects of their data. With our new documentation framework inspired by the AI Act, which recently entered into force, we encourage curators to reflect more critically on sources of noise and inaccuracy in their data. This is a necessary part of curation, a legal requirement for AI systems deployed in the medical domain, and, ultimately, a sign of a mature data ecosystem. We also encourage paper reviewers and data repository organizers to be more demanding about this aspect. It is worth noting that this trend would not have been highlighted by *Datasheets for Datasets*, where this information weights less than 2% of the questionnaire (as opposed to 20% of the Venus framework), or by the *Kaggle Dataset Usability Score*, where it is completely neglected.

Our results showed that only a few datasets among the ones considered reached a sufficient evaluation: only five datasets out of twelve attained a Venus score higher than 6 on 10. Two datasets stood out, reaching scores around 80%, both originating from greater Boston (Massachusetts, USA), a leading area for biomedical research [153]: the D7 GSE79209 dataset of bulk RNA-seq of patients diagnosed with lung cancer from Boston University and the D9 MIT-BIH dataset of electrocardiography signals of patients with arrhythmia from MIT. The former was released in 2017, and the latter in 1989, showing that good practices of data documentation can be independent of dataset age, despite growing awareness about this topic in recent years [11, 64]. Unlike other datasets, the curators of both D7 and D9 datasets paid great attention to patient privacy, data preparation, sources of inaccuracy, and noise. The high score of these datasets is likely favored by the release and maintenance standards of the specialized repositories where they are hosted. Of course, this factor is insufficient on its own, but it contributes to incentivizing thorough documentation by dataset curators.

### Added value and envisioned use

Our proposed Venus score can be of great advantage for several subjects. Data curators developing a new biomedical dataset can assess their documentation against the Venus score items, before releasing it publicly. Low-scoring items should prompt curators to enhance their documentation accordingly. Moreover, data users and researchers who find a dataset online can apply the Venus score to measure its reliability and trustworthiness, before performing their scientific analyses. Finally, watchdogs can use the Venus framework as a checklist for data quality requirements, including the main ones described in the AI Act.

Furthermore, we envision data repositories (Figshare, PhysioNet, Zenodo, UC Irvine ML Repository, and others) using our Venus score questions as a standard checklist for submitted datasets, making summaries of data trustworthiness immediately available to anyone downloading any dataset. Performing this in a centralized fashion across hundreds of datasets may be cumbersome; a dedicated comment section may encourage selected users to perform this evaluation and share it with the wider community, potentially prompting curators to review their documentation in an iterative fashion.

In sum, the Venus score contributes a compact set of questions that practitioners and researchers can use to reason about data trustworthiness and improve it. The numerical weighting scheme adopted in this article was chosen for two reasons: (1) it simplifies broad comparisons across diverse datasets and topics and (2) it provides an example of how this questionnaire can become a score; indeed, metrics and scores can introduce an element of gamification to data repositories and help improve the quality of shared data [154, 155].

### Limitations

Our work has some limitations. First, the proposed Venus score is not a stand-alone score for dataset selection. Complementary considerations are necessary to guide dataset adoption, including an evaluation of its representativeness for the context at hand. Second, answering the questionnaire and converting these answers into a score is subjective. This concern is partly mitigated if a precise weighting scheme is available. Additionally, the adoption of a single score to summarize a whole dataset may excessively aggregate a wealth of underlying information. Devoting attention to each question score (and verbose answer) remains fundamental to understanding the critical aspects of a dataset. Third, “Results” section focuses on publicly available datasets and their main documentation artifacts, influencing some of the trends we find. For example, a low Venus score in Table 4 signals that the key information about a dataset is not *readily* available on its release page and main accompanying article; in theory, this information may be available in less visible materials. Finally, we designed our Venus score for real data, not for simulated data. Simulated, synthetic, synthesized, and artificial data might need different criteria for quality and trustworthiness assessments.

### Future directions

Future work may consider expediting the calculation of our Venus score with semi-automated methods [156], since assessment in this work was fully manual and, therefore, time-consuming. Documentation sparsity should also be tackled: documentation analyses are complicated by the fact that information is often distributed among data repositories, *readme* files, websites, and publications. We envision an extension of the Venus score that rewards the compactness of available documentation and penalizes sparsity. Our Venus score will also be easily applicable to next-generation biomedical data types, such as whole-side bioimages [157–159] or single nucleus RNA sequencing (snRNA-seq) data [160]. Additionally, we will study how to integrate the Venus score into health standards, such as the HL7 fast healthcare interoperability resources (FHIR) protocol [161]. Finally, we envision exploiting selected components of the Venus score for assessing the readiness of biomedical datasets, based on the already-known data readiness levels [162–165] and similar concepts [166, 167].

## Supplementary information

Supplementary Material 1

Supplementary Material 2

## Data Availability

No datasets were generated or analysed during the current study.

## Abbreviations

AI: Artificial intelligence
BIT: Beth Israel Hospital
CITI: MIT Collaborative Institutional Training Initiative
CRAN: Comprehensive R Archive Network
CSV: Comma-separated values
DBT: Digital Breast Tomosynthesis
DICOM: Digital Imaging and Communications in Medicine
DOI: Digital object identifier
DREAM: Dialogue for Reverse Engineering Assessment and Methods
ECG: Electrocardiogram, electrocardiography
EEG: Electroencephalography
EHR: Electronic health record
EU: European Union
FAIR: Findability, accessibility, interoperability, and reusability
FHIR: Fast healthcare interoperability resources
GAAIN: Global Alzheimer’s Association Interactive Network
GEO: Gene Expression Omnibus
GSE: GEO Series
HL7: Health Level 7
kB: Kilobytes
IBD: Inflammatory bowel disease
ICU: Intensive care unit
IRB: Institutional review board
MAIDA: Medical AI Data for All
MIMIC: Medical Information Mart for Intensive Care
MIT: Massachusetts Institute of Technology
ML: Machine learning
NLP: Natural language processing
ODS: OpenDocument spreadsheet
PHI: Protected health information
RECORD: REporting of studies Conducted using Observational Routinely collected health Data
RNA-seq: RNA-sequencing
RNA: Ribonucleic acid
snRNA-seq: Single nucleus RNA sequencing
SRA: Sequence Read Archive
TCGA: The Cancer Genome Atlas
TCIA: The Cancer Imaging Archive
UC Irvine ML Repo: University of California Irvine Machine Learning Repository
URL: Uniform resource locator
XLSX: Microsoft Office Open XML
